# Mycobacteria Attenuate Nociceptive Responses by Formyl Peptide Receptor Triggered Opioid Peptide Release from Neutrophils

**DOI:** 10.1371/journal.ppat.1000362

**Published:** 2009-04-03

**Authors:** Heike L. Rittner, Dagmar Hackel, Philipp Voigt, Shaaban Mousa, Andrea Stolz, Dominika Labuz, Michael Schäfer, Michael Schaefer, Christoph Stein, Alexander Brack

**Affiliations:** 1 Klinik für Anaesthesiologie mit Schwerpunkt operative Intensivmedizin, Charité – Universitätsmedizin Berlin, Campus Benjamin Franklin, Berlin, Germany; 2 Klinik und Poliklinik für Anaesthesiologie, University of Würzburg, Würzburg, Germany; 3 Bereich Molekulare Pharmakologie und Zellbiologie, Charité – Universitätsmedizin Berlin, Campus Benjamin Franklin, Berlin, Germany; Johns Hopkins School of Medicine, United States of America

## Abstract

In inflammation, pain is regulated by a balance of pro- and analgesic mediators. Analgesic mediators include opioid peptides which are secreted by neutrophils at the site of inflammation, leading to activation of opioid receptors on peripheral sensory neurons. In humans, local opioids and opioid peptides significantly downregulate postoperative as well as arthritic pain. In rats, inflammatory pain is induced by intraplantar injection of heat inactivated *Mycobacterium butyricum*, a component of complete Freund's adjuvant. We hypothesized that mycobacterially derived formyl peptide receptor (FPR) and/or toll like receptor (TLR) agonists could activate neutrophils, leading to opioid peptide release and inhibition of inflammatory pain. In complete Freund's adjuvant-induced inflammation, thermal and mechanical nociceptive thresholds of the paw were quantified (Hargreaves and Randall-Selitto methods, respectively). Withdrawal time to heat was decreased following systemic neutrophil depletion as well as local injection of opioid receptor antagonists or anti-opioid peptide (i.e. Met-enkephalin, β-endorphin) antibodies indicating an increase in pain. *In vitro*, opioid peptide release from human and rat neutrophils was measured by radioimmunoassay. Met-enkephalin release was triggered by *Mycobacterium butyricum* and formyl peptides but not by TLR-2 or TLR-4 agonists. *Mycobacterium butyricum* induced a rise in intracellular calcium as determined by FURA loading and calcium imaging. Opioid peptide release was blocked by intracellular calcium chelation as well as phosphoinositol-3-kinase inhibition. The FPR antagonists Boc-FLFLF and cyclosporine H reduced opioid peptide release *in vitro* and increased inflammatory pain *in vivo* while TLR 2/4 did not appear to be involved. In summary, mycobacteria activate FPR on neutrophils, resulting in tonic secretion of opioid peptides from neutrophils and in a decrease in inflammatory pain. Future therapeutic strategies may aim at selective FPR agonists to boost endogenous analgesia.

## Author Summary

Inflammation of peripheral tissue can be caused by bacteria and is frequently accompanied by pain. Pain severity depends on the balance of enhancing (proalgesic) and decreasing (analgesic) mediators. Local endogenous pain control involves the release of opioid peptides from immune cells at the site of inflammation. These opioid peptides bind to opioid receptors on peripheral nerves and inhibit transmission of nociceptive impulses. We hypothesized that bacteria can directly stimulate immune cells to release opioid peptides and thereby decrease pain. In a rat model, inoculation of the paw with heat-inactivated *Mycobacterium butyricum* led to local inflammation and pain responses. Nociceptive thresholds were further decreased (i.e. pain was enhanced) following immune cell (i.e. neutrophil) depletion, local injection of anti-opioid peptide antibodies or opioid receptor antagonists. Immune cells recognize bacteria by toll-like and/or formyl peptide receptors. Previous research indicated that mycobacteria enhance nociceptive responses via toll like receptors-2 and -4. We now demonstrate that mycobacteria also activate formyl peptide receptors on neutrophils leading to opioid peptide release and the inhibition of such responses. Since bacteria can simultaneously induce the generation of pro- and analgesic mediators, our results might be a further explanation for differences in pain between individual patients following bacterial infections.

## Introduction

The four cardinal signs of inflammation are rubor (redness), calor (hyperthermia), dolor (pain/hyperalgesia) and functio laesa (impaired function). Bacteria and their components play a critical role in eliciting pain since inflammatory pain is significantly decreased in animals raised under germ free conditions [1]. Experimentally, inflammation can be elicited by local injection of heat inactivated *Mycobacterium butyricum* (“complete Freund's adjuvant”) resulting in spontaneous activity of nociceptive Aδ and C nerve fibers [2],[3]. Pain is elicited by proalgesic mediators including proinflammatory cytokines (tumor necrosis factor-α, interleukin-1β), bradykinin, and protons [2],[4]. Bacteria and their components are recognized by pattern recognition receptors including toll like receptors (TLR) as well as formyl peptide receptors (FPR). Peptidoglycan (a TLR-2 agonist), lipopolysaccharide (a TLR-4 agonist) and R-848 (a TLR-7 agonist) can elicit pain [5]–[7]. Furthermore, pain is decreased in TLR-4 deficient mice with bacterial cystitis [8] as well as in TLR-2 or -4 deficient mice with neuropathic lesions [9],[10]. In contrast to these pronociceptive effects of TLR agonists, FPR agonists were shown to decrease pain induced by formalin, but the underlying mechanism remained unclear [11].

The intensity of inflammatory pain is not only dependent on proalgesic mediators, but is counteracted by endogenous analgesic mediators including opioid peptides [12]. Both neutrophils and monocytes contain opioid peptides (Met-enkephalin and β-endorphin) and they are the predominant leukocyte subpopulations during the first 4 days of complete Freund's adjuvant-induced inflammation [13]–[15]. Opioid peptides are released, bind to opioid receptors on peripheral sensory neurons and induce analgesia (i.e. decrease of inflammatory pain). Releasing agents such as hormones (e.g. corticotrophin releasing hormone [16]) or chemokines (CXCL2/3) [17],[18] trigger opioid release from leukocytes *in vitro* and induce opioid-mediated analgesia *in vivo*. In the experimental model, peripheral endogenous opioid analgesia requires injection of these releasing agents at the site of inflammation. This effect is short lasting (max. 10 min) making this approach not attractive for the clinical setting. Interestingly, however, both clinical [19] and experimental studies [20] indicate that opioid peptides might be continuously released at the site of surgery or experimental inflammation and decrease inflammatory pain. At present, it is unclear how continuous release is regulated.

It is tempting to speculate that *Mycobacterium butyricum*, as a component of complete Freund's adjuvant, triggers opioid peptide release from leukocytes and, thereby, induces analgesia. Mycobacteria activate both TLR [21],[22] and FPR [23] that are expressed on neutrophils and monocytes/macrophages [24]–[26]. Of the ten known TLR, mycobacteria predominantly interact with TLR-2 and TLR-4 through lipoproteins and lipomannans [21],[27]. TLR and/or FPR stimulation of neutrophils induce L-selectin shedding, enhanced CD11b expression and/or release of reactive oxygen species [28]–[31]. FPRs are coupled to G_i_ proteins [24] and receptor activation triggers intracellular signaling through phospholipase C, diacylglycerol, inositol phosphates and Ca^2+^ mobilization from intracellular stores, as well as activation of phosphoinositol-3 kinase (PI3K) [32]. In contrast, TLR activation induces coupling to an adapter protein, MyD88, and stimulation of several intrinsic kinases including interleukin-1 receptor accessory protein kinase leading to NF-κB activation.

In this study we examined whether heat inactivated *Mycobacterium butyricum* triggers opioid peptide release from rat and human neutrophils and monocytes and whether this requires FPR and/or TLR activation. We further studied the downstream signaling mechanisms of receptor activation. Finally, we tested the *in vivo* functional relevance of FPR agonist- and of *Mycobacterium butyricum*-induced opioid peptide release as an endogenous pathway of pain control in complete Freund's adjuvant-induced inflammation. We found that *Mycobacterium butyricum* induced opioid peptide release from neutrophils through FPR but not TLR stimulation. Mycobacterium-triggered opioid peptide release required intracellular calcium mobilization and PI3K activation. *In vivo* this mechanism decreased inflammatory pain mainly in early inflammation.

## Results

### Inflammatory pain is attenuated by tonic opioid peptide release from neutrophils

**Figure 1:**
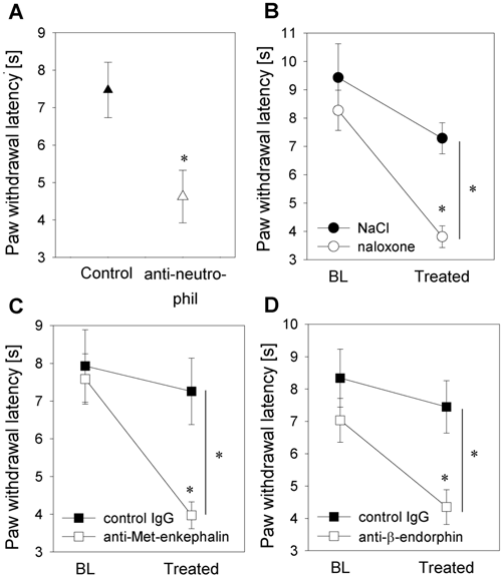
Neutrophils attenuate inflammatory pain by local opioid peptide release. [A] In a rat model, inflammation was induced by intraplantar injection of heat-inactivated *Mycobacterium butyricum* (complete Freund's adjuvant). Prior to induction of inflammation, rats were pretreated with i.v. anti-neutrophil serum (open triangles). Control animals received non-immune rabbit serum (filled triangle). Two hours after complete Freund's adjuvant inoculation thermal nociceptive thresholds (i.e. paw withdrawal latency) were significantly decreased in neutropenic rats (n = 6–8; * p<0.05, t-test). [B, C] To examine the role of endogenous opioids in local inflammatory pain control, rats with complete Freund's adjuvant-induced inflammation were intraplantarly injected with the opioid receptor antagonist naloxone (0.56 ng, control solvent only; open and filled circle respectively; baseline, BL [B]) (n = 9–14), with an anti-opioid peptide antibody (i.e. anti-Met-enkephalin, 1.25 µg, or anti-β-endorphin, 2 µg) or with control IgG; open and filled squares, respectively [C, D]) and nociceptive thresholds were determined. Nociceptive thresholds were significantly decreased following both treatments (* p<0.05 all one way ANOVA, Dunn's method). Data are presented as means±SEM.

Intraplantar complete Freund's adjuvant injection containing *Mycobacterium butyricum* resulted in a significant decrease in thermal nociceptive thresholds (paw withdrawal latency) in comparison to noninflamed contralateral paws indicating inflammatory pain (paw withdrawal latency in inflamed paws 8.9±2.4 s vs. paw withdrawal latency in noninflamed contralateral paws 19.3±2.0 s). To assess whether pain after intraplantar complete Freund's adjuvant injection was affected by infiltrating neutrophils at the site of inflammation, systemic neutrophil depletion was performed. Consistent with previous findings, neutrophils in the circulation and at the site of complete Freund's adjuvant-induced paw inflammation were reduced by >90% while monocytes/macrophages were unaffected [14],[17]. Neutropenia was associated with significantly lower thermal nociceptive thresholds (paw withdrawal latency; Fig. 1A). Since neutrophils were previously shown to contain and release Met-enkephalin and β-endorphin upon stimulation (e.g. by CXCR2 ligands) [17], we examined whether tonic opioid release attenuates inflammatory pain. Intraplantar injection of naloxone, an opioid receptor antagonist (Fig. 1B), anti-Met-enkephalin or anti-β-endorphin antibodies (Fig. 1C, D) significantly reduced thermal nociceptive thresholds for up to 30 min (data not shown). No changes were seen after subcutaneous application of the same dose of naloxone, anti-Met-enkephalin or anti-β-endorphin antibody into a skin fold of the back, indicating the absence of systemic effects (data not shown). Taken together, these data suggest that neutrophils tonically secrete opioid peptides and, thereby, significantly reduce inflammatory pain.

### Mycobacteria can trigger opioid peptide release from neutrophils but not from monocytes

**Figure 2:**
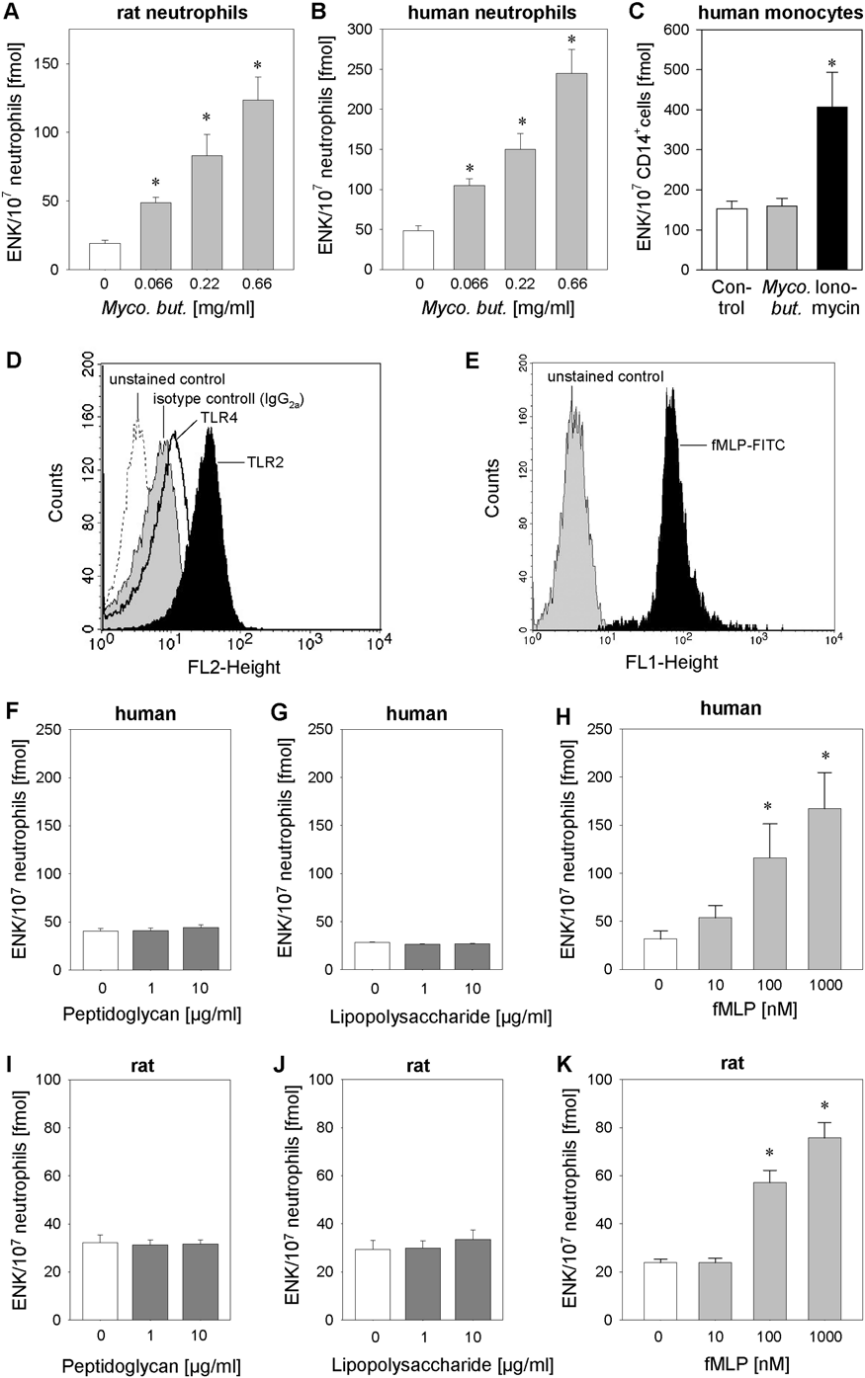
Opioid peptide release from neutrophils is triggered by mycobacteria and FPR agonists but not by TLR-2 or TLR-4 agonists. [A–C] Rat and human neutrophils as well as CD14^+^ human monocytes were incubated with heat-inactivated *Mycobacterium butyricum* (*Myco. but.*), and Met-enkephalin (ENK) release was quantified by radioimmunoassay (n = 7–13 * p<0.05, one way RM ANOVA, Student-Newman-Keuls Method). [D, E] Expression of TLR-2, TLR-4 [D] and FPR [E] was determined on human neutrophils by flow cytometry (dotted line: unstained control, grey histogram: isotype control, black line: anti-TLR-2-PE, black histogram: fMLP-FITC or anti-TLR-4-PE). [F–H] Human (n = 7–16) and [I–K] rat neutrophils (n = 7–21) were stimulated with the TLR-2 agonist peptidoglycan, the TLR-4 agonist lipopolysaccharide or the FPR agonist fMLP, and Met-enkephalin (ENK) release was measured in the supernatant (* p<0.05; one way RM ANOVA, Student-Newman-Keuls Method). Data are presented as means±SEM.

We hypothesized that *Mycobacterium butyricum* might directly trigger opioid peptide release. Incubation of human and rat neutrophils with *Mycobacterium butyricum* resulted in dose-dependent release of Met-enkephalin (Fig. 2A, B). In contrast, no release of Met-enkephalin after *Mycobacterium butyricum* stimulation was observed in human blood monocytes following short term (7 min; Fig. 2C) or long term stimulation (up to 2 h; data not shown) although monocytes express FPR [33],[34] and TLR [35]. However, human monocytes were able to secrete Met-enkephalin after stimulation with ionomycin, a calcium ionophore, as a positive control. Similarly, human and rat neutrophils released β-endorphin upon stimulation with *Mycobacterium butyricum* but human monocytes only secreted β-endorphin after ionomycin stimulation (Fig. S1).

### Stimulation of FPR but not of TLR-2 or TLR-4 elicits opioid peptide secretion from neutrophils in vitro and opioid-mediated analgesia *in vivo*

Since mycobacteria activate TLR-2 and TLR-4 on neutrophils [22],[27],[28], we hypothesized that agonist stimulation of these receptors might induce opioid peptide release. In line with previous studies [26], both TLR-2 and TLR-4 were expressed on human neutrophils as measured by flow cytometry (Fig. 2D). However, no Met-enkephalin release was seen after stimulation of TLR-2 with peptidoglycan [36] or stimulation of TLR-4 with lipopolysaccharide [36] in human or rat neutrophils (Fig. 2F, G, I, J). Mycobacteria also contain formylated peptides activating FPR [23],[37]. In accordance with previous studies [38] FPR were expressed on human blood neutrophils (Fig. 2E). Incubation of human and rat neutrophils with formyl-Met-Leu-Phe (fMLP), an FPR agonist, resulted in dose-dependent release of Met-enkephalin (Fig. 2H, K). No release of Met-enkephalin was observed after fMLP stimulation of human monocytes (data not shown). Similar results were obtained for release of β-endorphin from human and rat neutrophils (Fig. S1).

**Figure 3:**
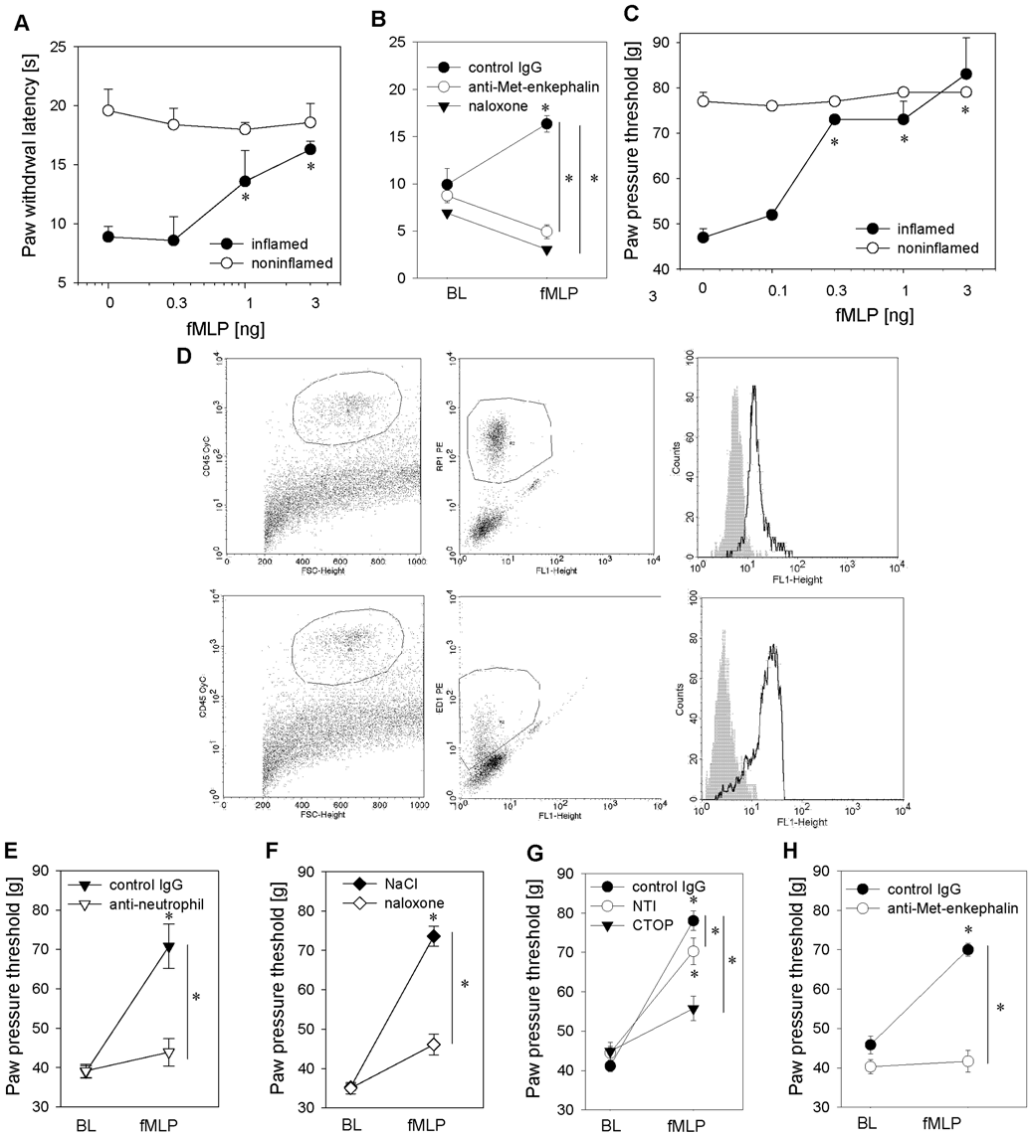
Formyl peptides inhibit inflammatory pain through release of opioid peptides from neutrophils. [A, C] Rats received intraplantar injections of fMLP into inflamed paws (2 h post complete Freund's adjuvant: filled circles; noninflamed paw: open circles). Paw withdrawal latency [A] or paw pressure thresholds [C] were determined 5–7 min after injections (n = 5–6, * p<0.05 one way ANOVA, Duncan's and Dunnett's method, respectively as well as p<0.001 linear regression analysis for dose-dependency of fMLP-induced analgesia). [B] Two hours post complete Freund's adjuvant rats received intraplantar fMLP (3 ng) together with either control IgG (filled circles), antibody against Met-enkephalin (open circles) or naloxone. Paw withdrawal latency was measured 5–7- min thereafter (baseline, BL; n = 6;* p<0.05 one way ANOVA, Duncan's method). [D] Subcutaneous paw tissue was analyzed for FPR expression by flow cytometry. Cells were first gated on CD45^+^ hematopoetic cells (left panel) followed by gating on RP1-PE^+^ neutrophils (upper middle panel) or ED1-PE^+^ macrophages (lower middle panel). Staining with fMLP-FITC (solid lanes) was analyzed in comparison to unstained controls (grey histograms) (right panel). [E] Systemic neutrophil depletion by intravenous injection of anti-neutrophil serum 18 h before induction of inflammation (anti-neutrophil, open triangles; control: rabbit IgG, closed triangles; n = 6, * p<0.05 one way RM ANOVA, Duncan's method) abolished fMLP-induced analgesia. [F–H] Similarly, concomitant intraplantar injection of fMLP with either the opioid receptor antagonist naloxone ([F] 0.56 ng, open diamond, control: solvent, filled diamond; n = 6, * p<0.05 one way ANOVA, Dunnett's method), CTOP ([G] 50 µg, filled triangles), NTI ([G] 20 µg, open circles; n = 6, * p<0.05 one way ANOVA, Student–Newman-Keuls) or anti–Met-enkephalin antibody ([H] 1.25 µg open circles; control: rabbit IgG, filled circles; n = 4–5) resulted in significant inhibition of analgesia (* p<0.05 one way ANOVA, Duncan's method). All data are means±SEM.

We next evaluated whether fMLP can induce analgesia in rats with complete Freund's adjuvant -induced hindpaw inflammation. Complete Freund's adjuvant injection resulted in inflammatory pain measured by a significant decrease in both mechanical (paw pressure threshold) and thermal nociceptive thresholds (paw withdrawal latency) in comparison to noninflamed contralateral paws (Fig. 3A, C). fMLP injected intraplantarly into inflamed hindpaws elicited significant and dose-dependent analgesia as indicated by a rise in mechanical and thermal nociceptive thresholds (Fig. 3A, C). fMLP-induced analgesia peaked at 5 min, was still elevated after 10 min and returned to baseline after 20 min (data not shown). Higher doses of fMLP were needed to reverse thermal nociceptive threshold in inflamed hind paws (Fig. 3A).

FPR is expressed on neutrophils as well as monocytes/macrophages [34]. We detected FPR expression on CD45^+^RP-1^+^ neutrophils as well as CD45^+^ED1^+^ macrophages isolated from the inflamed paw (Fig. 3D). fMLP-induced analgesia was abolished by selective systemic neutrophil depletion (Fig. 3E), by peripherally selective blockade of mu-opioid receptors (naloxone, Fig. 3B, F, CTOP, Fig 3G) or by neutralization of opioid peptides (i.e. anti Met-enkephalin antibodies, Fig. 3B, H). Blockade of delta-opioid receptors partially but significantly reduced nociceptive thresholds after fMLP injection (naltrindole, Fig. 3G).

**Figure 4:**
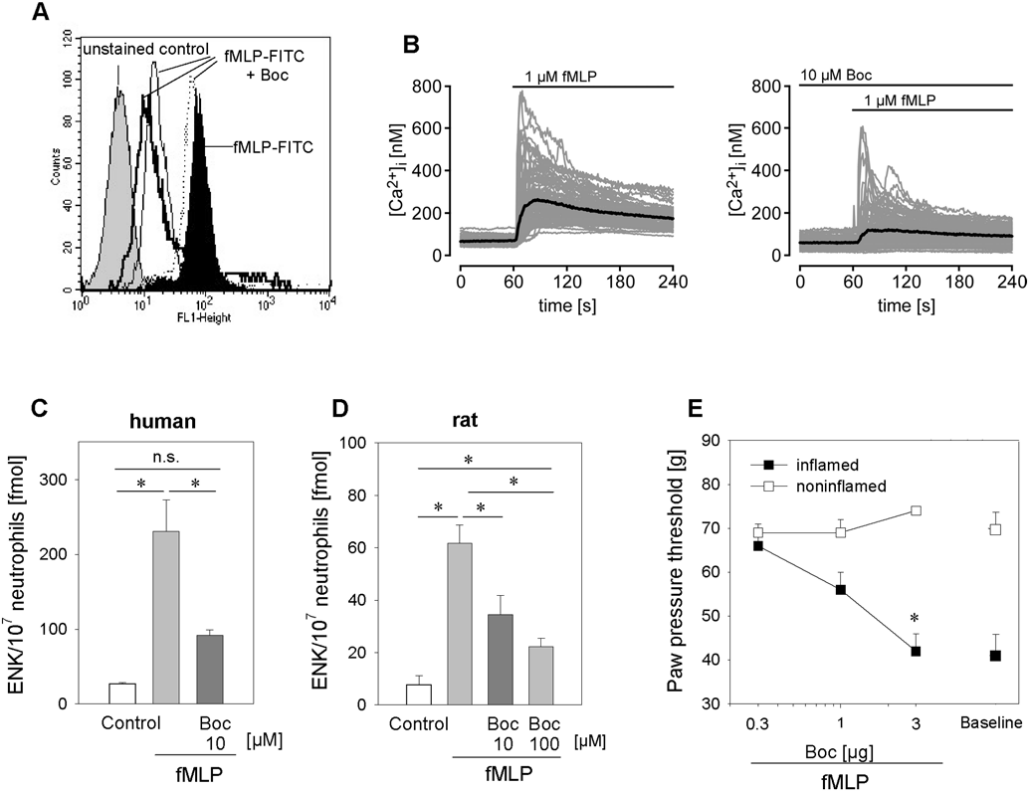
fMLP-induced opioid peptide release in vitro and analgesia *in vivo* are dependent on activation of the FPR. [A] FPR staining of human neutrophils with fMLP-FITC (black histogram) is dose-dependently inhibited by the FPR antagonist Boc-FLFLF (Boc, dotted line: 0.1 µM, thin black line: 1 µM, thick black line: 10 µM). The unstained control is shown in the gray histogram. [B] Neutrophils were loaded with Fura-2 and changes in [Ca^2+^]_i_ were analyzed after addition of fMLP (left panel) and after preincubation with Boc-FLFLF and subsequent stimulation with fMLP (right panel). Boc-FLFLF also blocked the fMLP-triggered release of Met-enkephalin (ENK) from human [C] and rat [D] neutrophils (n = 5–9; both * p<0.05 one way RM ANOVA, Student-Newman-Keuls Method). [E] *In vivo*, intraplantar injection of Boc-FLFLF blocked fMLP-induced analgesia (0.3 ng fMLP) in the inflamed paw (filled squares; noninflamed paws: open squares). Baseline hyperalgesia prior to fMLP injection is shown for comparison (n = 6; * p<0.05 one way ANOVA, Duncan's method, and p<0.001 linear regression analysis for dose-dependency of FPR blockage). Data are means±SEM.

To verify the involvement of FPR we employed two FPR inhibitors, N-t-Boc-Phe-D-Leu-Phe-D-Leu-Phe (Boc-FLFLF) and cyclosporine H [31],[39],[40]. Boc-FLFLF dose-dependently reduced fMLP-FITC binding to human neutrophils (Fig. 4A). In parallel, fMLP-triggered elevation of intracellular calcium in human neutrophils was inhibited by preincubation with Boc-FLFLF (Fig. 4B) or cyclosporine H (data not shown). Met-enkephalin release from human neutrophils was completely blocked by preincubation with 10 µM Boc-FLFLF (Fig. 4C) and was reduced by 62 and 72% after preincubation with 10 or 100 µM cyclosporine H, respectively (Table 1). In rat neutrophils, higher doses of FPR inhibitors were necessary. The fMLP-induced Met-enkephalin release was inhibited by 69% using 100 µM Boc-FLFLF (Fig. 4D) and by 44% using 100 µM cyclosporine H (Table 1). To test these FPR antagonists *in vivo* we intraplantarly injected rats with complete Freund's adjuvant and either antagonist together with fMLP. Both FPR antagonists dose-dependently blocked fMLP-induced analgesia (Fig. 4E, Table 1).

**Table 1:** Effect of the FPR antagonist cyclosporine H (CsH) on fMLP-induced Met-enkephalin release from neutrophils and on fMLP-induced antinociception.

Opioid peptide release				
Met-enkephalin [fmol/10^7^ neutrophils]	Control	fMLP 1 µM	CsH 10 µM fMLP 1 µM	CsH 100 µM fMLP 1 µM
Human neutrophils	66±6	309±20*	158±19* §	134±4* §
Rat neutrophils	33±5	207±18*	n.d.	130±8* §

Pain Behaviour					
Paw pressure threshold [g]	Baseline	fMLP 0.3 µg	CsH 0.09 µg fMLP 0.3 µg	CsH 0.9 µg fMLP 0.3 µg	CsH 9 µg fMLP 0.3 µg
Inflamed	38.6±1.6	80.7±3.8	71.3±2.7	50.5±1.8¶	60.4±1.8¶
Noninflamed	67.6±2.2	71.4±1.5	71.4±1.4	70.7±2.8	69.1±2.2

*: p<0.05 (one way repeated measures ANOVA, Student-Newman-Keuls Method) versus control medium.

**§:** p<0.05 versus fMLP (human: n = 13–25, rat: n = 14–22; n.d. = not determined).

**¶:** p<0.05 (one way ANOVA, Duncan's Method) versus fMLP (n = 6).

### Mycobacteria stimulate FPR-dependent intracellular calcium mobilization and PI3K-dependent release of opioid peptides

**Figure 5:**
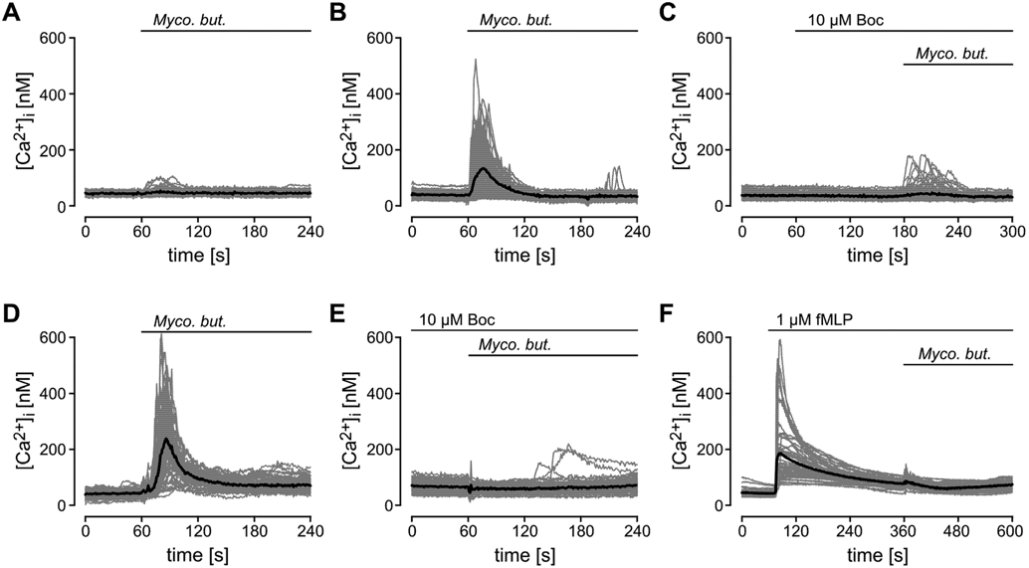
Mycobacterium butyricum-triggered intracellular calcium elevation is FPR dependent. Mock [A] or human FPR [B,C] transfected HEK293 cells or human neutrophils [D–F] were loaded with Fura-2. Changes in [Ca^2+^]_i_ were analyzed after addition of *Mycobacterium butyricum* (*Myco. but.*) at 60 s in the presence [C, E] or absence [B,D] of 10 µM Boc-FLFLF (Boc). [F] Impact of fMLP stimulation on subsequent stimulation with *Mycobacterium butyricum* in human neutrophils.

*Mycobacterium butyricum* triggered intracellular Ca^2+^ mobilization in FPR -, but not in mock-transfected human embryonic kidney (HEK) 293 cells (Fig. 5A, B). This was blocked by preincubation with the FPR antagonist Boc-FLFLF (Fig. 5C) or cyclosporine H (data not shown). Similar changes were observed in human neutrophils (Fig. 5D–F). Acute receptor desensitization was observed because stimulation of human neutrophils with fMLP almost completely abolished subsequent stimulation with *Mycobacterium butyricum* (Fig. 5F).

**Figure 6:**
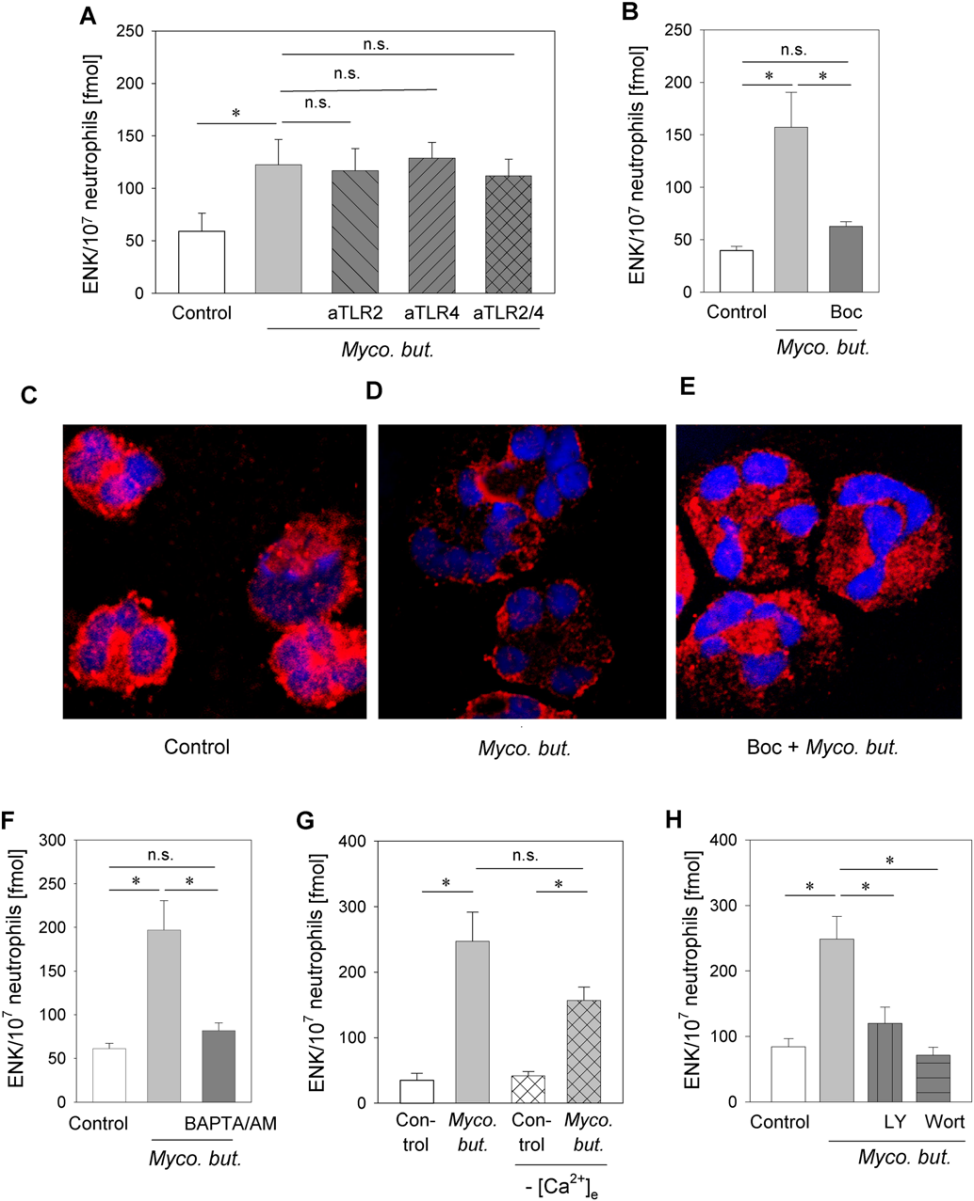
Mycobacterium butyricum-induced opioid peptide release and translocation of Met-enkephalin-containing granules from human neutrophils requires FPR stimulation, intracellular Ca^2+^ mobilization and PI3K activation. [A, B] Met-enkephalin release induced by *Mycobacterium butyricum* (*Myco. but.* 0.66 mg/ml) was analyzed after preincubation with anti-TLR-2 and anti-TLR-4 ([A], anti-TLR2 or 4 both 10 µg/ml, n = 10–12), or the FPR antagonist Boc-FLFLF ([B], Boc 10 µM; n = 7–8, * p<0.05, both one way RM ANOVA, Student-Newman-Keuls Method). [C–E] Cytospins from freshly isolated human neutrophils previously incubated with *Mycobacterium butyricum* in the presence [F] or absence [E] of the FPR antagonist Boc-FLFLF were stained with anti-Met-enkephalin Ab (red; [C] solvent control only). Original magnification ×63. [F–H] *Mycobacterium butyricum*-induced opioid peptide release was prevented by the intracellular Ca^2+^ chelator BAPTA/AM (100 µM, [F]; n = 8) but not dependent on extracellular Ca^2+^ ([G], n = 7–14, no [Ca^2+^]_e_ crosshatched bars). [H] *Mycobacterium butyricum*-induced opioid peptide release was also blocked by the PI3K-inhibitors LY294002 (LY, 100 µM) and wortmannin (wort, 100 nM, n = 8–10). All experiments * p<0.05 one way RM ANOVA, Student-Newman-Keuls Method. Data are means±SEM.

We further examined the role of TLR and FPR in mycobacterial stimulation of opioid peptide release. No change in *Mycobacterium butyricum*-triggered opioid peptide release was seen after blockade with single or combined anti-TLR-2 and anti-TLR-4 (Fig. 6A) while the addition of two FPR antagonists, Boc-FLFLF and cyclosporine H, resulted in an 80% and 74% reduction of Met-enkephalin release, respectively (Fig. 6B and Table 2).

**Table 2:** Effect of the FPR antagonist cyclosporine H (CsH) on *Mycobacterium butyricum*-(*Myco. but.*) induced Met-enkephalin release from human and rat neutrophils and on nociceptive thresholds in complete Freund's adjuvans induced hindpaw inflammation.

Opioid peptide release				
Met-enkephalin [fmol/10^7^ neutrophils]	Control	*Myco. but.*	CsH 10 µM *Myco. but.*	CsH 100 µM *Myco. but.*
Human neutrophils	39.3±4.2	143.8±12.1*	66.7±8.3* §	n.d.
Rat neutrophils	18.5±2.2	239.3±11.9*	n.d.	148.6±7.3* §

Pain Behaviour				
Paw withdrawal latency [s]	0 min	15 min post injection	30 min post injection	60 min post injection
Cyclosporine H (9 µg i.pl.)	7.1±0.3	4.6±0.6¶	4.2±0.5¶	4.1±0.6¶
Solvent	6.7±0.6	7.2±0.7	6.2±0.5	6.2±0.4

*: p<0.05 (one way repeated measures ANOVA, Student-Newman-Keuls method) vs. Control medium.

**§:** p<0.05 versus *Myco. but.* (0.66 mg/ml) (human: n = 16–18; rat: n = 7–14; n.d. = not determined).

**¶:** p<0.05 versus 0 min (one way repeated measures ANOVA, Duncan's method, n = 10–16).

Activation of neutrophils leads to the translocation of primary granules to the plasma membrane to allow for release [18],[41]. Unstimulated neutrophils exhibited a homogeneous cytoplasmic granular staining for Met-enkephalin (Fig. 6C). Following stimulation with *Mycobacterium butyricum*, large aggregates of Met-enkephalin-containing granules formed in submembranous regions and overall staining was significantly reduced as a sign of degranulation (Fig. 6D). Preincubation with Boc-FLFLF inhibited *Mycobacterium butyricum*-induced translocation of Met-enkephalin-containing granules to the cell membrane (Fig. 6E).

Elevation of intracellular Ca^2+^ is required for opioid peptide release [17]. FPR is known to signal through G_i_ proteins stimulating phospholipase C leading to mobilization of Ca^2+^ from intracellular stores and to activation of PI3K [24]. Both chelation of intracellular calcium by BAPTA/AM (Fig. 6F) as well as pretreatment with the PI3K inhibitors (LY294002 and wortmannin, Fig. 6H) prevented *Mycobacterium butyricum*-induced opioid peptide release. In contrast, *Mycobacterium butyricum*-induced Met-enkephalin release was independent of extracellular calcium (Fig. 6G).

### Blockade of FPR *in vivo* increased inflammatory pain by inhibiting tonic opioid peptide release

**Figure 7:**
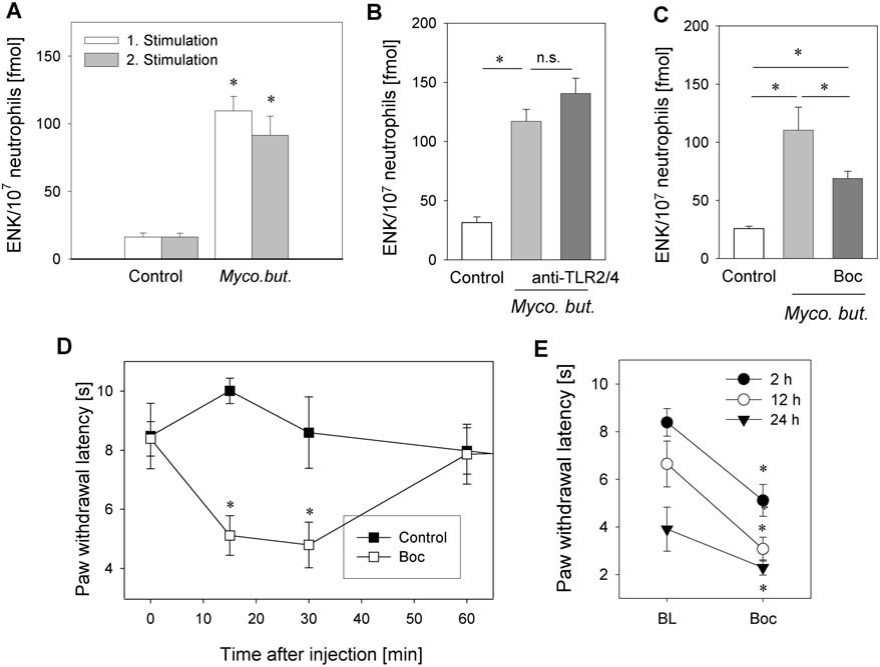
Tonic opioid peptide release through FPR stimulation reduces inflammatory pain. [A] Rat neutrophils were repeatedly stimulated with *Mycobacterium butyricum* (*Myco. but.* 0.66 mg/ml) and Met-enkephalin (ENK) content was determined in supernatants (n = 12–16; * p<0.05, one way RM ANOVA, Student-Newman-Keuls Method). [B, C] *Mycobacterium butyricum*-induced Met-enkephalin release was unaltered by anti-TLR-2 and -TLR-4 antibodies ([B], anti-TLR-2/4 both 10 µg/ml, n = 8–19), but was significantly reduced by FPR antagonist Boc-FLFLF ([C], Boc: 100 µM, n = 7–11, both * p<0.05 one way RM ANOVA, Student-Newman-Keuls Method. [D] Rats with 2 h complete Freund's adjuvant inflammation were intraplantarly injected with Boc-FLFLF (Boc, 3 µg, [D], n = 5–6) and nociceptive thresholds were determined (*p<0.05 one way RM ANOVA, Duncan Method). Data are means±SEM. [E] Rats (n = 6) with 2, 12 and 24 h complete Freund's adjuvant inflammation were treated with 3 µg Boc intraplantarly and paw withdrawal latency was measured afterwards (* p<0.05, Wilcoxon Signed Rank test compared to baseline (BL)).

Tonic opioid peptide release from neutrophils *in vivo* would require that stimulation with *Mycobacterium butyricum* does not completely empty all stores of opioid peptides after a single stimulation. To test this we repeatedly stimulated neutrophils with *Mycobacterium butyricum in vitro*. After the second stimulation with *Mycobacterium butyricum* we detected the same amount of Met-enkephalin in the supernatant (Fig. 7A). In addition, we compared Met-enkephalin release after *Mycobacterium butyricum* with maximal stimulation elicited by the calcium ionophore ionomycin. *Mycobacterium butyricum* only mobilized 19% of the ionomycin-induced Met-enkephalin release (data not shown).

Similar to human neutrophils, mycobacterium-triggered Met-enkephalin release from rat neutrophils was unaltered by TLR-2/4 blockade (Fig. 7B) but was inhibited by preincubation with Boc-FLFLF by 49% (Fig. 7C) and with cyclosporine H by 41% (Table 2).

To test whether formyl peptides might be involved in tonic analgesia through the release of opioid peptides *in vivo*, we treated rats with complete Freund's adjuvant-induced inflammation with intraplantar injection of Boc-FLFLF (Fig. 7D) or cyclosporine H (Table 2) using optimal doses determined in prior dose response experiments (Fig. 4E and Table 1). Both treatments significantly lowered thermal nociceptive thresholds for up to 30 min indicating increased inflammatory pain. Boc-FLFLF treatment significantly reduced thermal nociceptive thresholds also after 12 and 24 h complete Freund's adjuvant inflammation (Fig. 7E). However, the effect was less prominent after 24 h (1.6 s difference vs. 3.3 s difference at 2 h of complete Freund's adjuvant inflammation). Basal nociceptive threshold progressively fell during inflammation indicating that hyperalgesia increased over time (Fig. 7E).

## Discussion

Bacteria have long been believed to trigger inflammatory pain by activating immune cells of the innate immune system. In this study, we demonstrate that bacteria simultaneously decrease pain by stimulating tonic release of endogenous opioid peptides like Met-enkephalin and β-endorphin at the site of inflammation. *In vitro*, heat inactivated *Mycobacterium butyricum* triggers opioid peptide release from neutrophils, but not from monocytes. This requires activation of FPR as well as intracellular Ca^2+^ mobilization and PI3K activation, while TLR-2 and -4 do not seem to be involved. These pathways are relevant *in vivo* since pain increases if FPR are blocked at the site of complete Freund's adjuvant-induced inflammation.

Local injection of *Mycobacterium butyricum* (i.e. complete Freund's adjuvant) induces inflammatory pain as demonstrated by a decrease in thermal and mechanical nociceptive thresholds. The thermal pain threshold is further decreased by prior systemic neutrophil depletion (Fig. 1A). Intuitively, one would expect that the removal of neutrophils reduces the inflammatory reaction. However neutrophil depletion in CFA inflammation does not significantly change paw volume or local prostaglandin E2 production but leads to a reduction in total IL-1ß content. Despite the neutrophil depletion nociceptive thresholds were not decreased [42]. Similarly, selective neutrophil recruitment by intraplantar CXCL2/3 injection does not elicit signs of inflammation or lowered nociceptive thresholds [42]. This suggests that neutrophils contribute modestly to the inflammatory reaction and are more important for the inhibition than the generation of pain. In previous studies, neutrophils were shown to be the major opioid peptide containing leukocyte population in early inflammation (within 24 h of injection) while monocytes/macrophages are predominant in later stages [13],[14],[17],[18]. Opioid peptide release requires a stimulus such as cold water swim [43] or intraplantar injection of corticotrophin releasing hormone, cytokines (e.g. interleukin-1) [44] or chemokines (CXCL2/3) [17],[18]. Although the resultant analgesia is potent, it only lasts for 5–10 min. Thus, it has been an open question whether there is a biological role of this system under basal inflammatory conditions. In line with previous studies in postoperative pain in humans [19], we now demonstrate that peripherally mediated opioid analgesia is active under basal conditions in complete Freund's adjuvant-induced inflammation. Local injection of the opioid receptor antagonist naloxone, anti-Met-enkephalin or anti-β-endorphin antibodies resulted in a further decrease in thermal nociceptive thresholds and, thus, enhanced inflammatory pain (Fig. 1B–D). These reductions were detectable when measuring thermal (i.e. Hargreaves method) but not mechanical nociceptive thresholds (i.e. Randall Selitto test) presumably because of the limited sensitivity of the latter test (data not shown). We next identified the molecular mechanisms responsible for tonic opioid peptide release.

Complete Freund's adjuvant contains heat inactivated *Mycobacterium butyricum*. *In vitro*, neutrophil but not monocyte stimulation with *Mycobacterium butyricum* resulted in a significant and dose-dependent release of Met-enkephalin (Fig. 2A–C). To further delineate the molecular pathways, we first explored TLR-2 and TLR-4, the major receptors transmitting the signals of mycobacteria [22],[28]. Expression of TLR-2 and TLR-4 on neutrophils has been shown on mRNA and protein levels [29],[45] as well as functionally through stimulation with lipopolysaccharide or peptidoglycan [29],[30],[45]. We could not detect Met-enkephalin or β-endorphin release after selective TLR activation (Fig. 2 and Fig. S1) despite receptor expression. In line with the lack of opioid peptide release after TLR-2 or TLR-4 stimulation, the *Mycobacterium butyricum*-induced release of Met-enkephalin could not be blocked by single or combined TLR-2/4 blockade (Fig. 6A, 7B). Antibodies against TLR-2 and -4, although widely used, have mostly shown partial inhibitory effects [46]–[52]. Therefore, the additional involvement of TLR cannot completely be excluded and would have to be studied in TLR knockout mice. Other studies demonstrated that TLR can induce production of reactive oxygen species [29],[30] and PI3K-dependent tumour necrosis factor-α and CXCL2/3 secretion [53]. Costimulation with fungi and TLR-4 agonists enhances secretion of primary granules, whereas TLR-2 agonists increase tertiary granule secretion. In contrast to our study, neutrophils were stimulated for 4 h, TLR agonists were not tested in the absence of fungal products and the TLR-2/4-induced increase in release was modest in this study [54]. In line with these data, short term TLR stimulation (i.e. minutes) in the absence of costimulators does not induce substantial granule release from neutrophils [55]. Since opioid peptides are stored in primary granules in neutrophils [18] and can be released within minutes, our results are in accordance with the current literature.

Mycobacteria contain formyl peptides [23], which are released during bacterial lysis [25]. Activation of human neutrophils with mycobacteria or fMLP induced a more than fivefold increase in Met-enkephalin secretion (Fig. 2B and 2H, respectively). Both fMLP- and *Mycobacterium butyricum*-induced opioid peptide release was blocked by the specific FPR antagonists Boc-FLFLF (Fig. 4C, 6B) and cyclosporine H (Tables 1 and 2). Previous studies delineated signaling requirements for fMLP-triggered release [24]. We now demonstrate that the same signaling pathways are activated following mycobacterial stimulation. Specifically, we found that mycobacteria and fMLP triggered intracellular Ca^2+^ mobilisation in neutrophils (Fig. 4D) and in HEK293 cells transfected with human FPR but not in Mock-transfected cells (Fig. 4B). *Mycobacterium butyricum* did not induce intracellular Ca^2+^ mobilisation if cells were pretreated with fMLP demonstrating acute FPR desensitisation. Furthermore, opioid peptide release was dependent on intracellular calcium mobilisation as well as PI3K activation (Fig. 6F–H). Both are known to be downstream signals of FPR but not TLR activation. To underline the *in vivo* relevance of our findings we demonstrate that formyl peptides (i.e., fMLP) can induce analgesia in complete Freund's adjuvant-induced inflammation, mediated through mu- and delta opioid receptors (Fig. 3B,G) and that local injection of FPR antagonists significantly impairs local endogenous pain control (Fig. 7 and Table 2). FPR mediated endogenous pain control was seen for up to 24 h, but became less prominent because baseline thermal nociceptive threshold decreased during the time course of inflammation (Fig. 7E). This is consistent with the number of infiltrating neutrophils in complete Freund's adjuvant inflammation [15]. Constant recruitment of neutrophils from the circulation as well as submaximal stimulation could account for the tonic release of opioid peptides without FPR desensitisation. Indeed, only around 20% of the total opioid peptide content from neutrophils was secreted during the first simulation, and repetitive stimulation of neutrophils allowed for repeated release of Met-enkephalin if the stimulus was washed away in between (Fig. 7A). In conclusion, intracellular stores of opioid peptides seem to contain enough opioid peptides to permit tonic release after repetitive stimulation with *Mycobacterium butyricum*.

In our studies fMLP- as well as *Mycobacterium butyricum*-induced Met-enkephalin release could be completely blocked by Boc-FLFLF and partially blocked by cyclosporine H in human neutrophils (Fig. 4C, 6B and Tables 1 and 2). In rats, both Boc-FLFLF and cyclosporine H were only partially effective and higher doses were required (Fig. 4D, 7C and Tables 1 and 2). While species differences cannot be fully excluded, they appear unlikely. In contrast to mice [56], human and rat FPR show a comparable and high affinity for fMLP [57]. The two FPR antagonists Boc-FLFLF and cyclosporine H are functional in rodents since they significantly reduce monocyte and neutrophil recruitment in murine pneumococcal pneumonia [58],[59] and impair the protective effect of fMLP on infarct size in a rat model of ischemia reperfusion injury [60]. Alternatively, differences in activation state of neutrophils might be important. We performed our experiments in purified human peripheral blood neutrophils from healthy volunteers, while rat neutrophils were obtained by sterile peritonitis, which induces significant preactivation [61]. Therefore it is conceivable that other receptors (e.g. chemokine receptors [62]) need to be blocked in addition to completely abolish opioid peptide secretion. This view is supported by our previous study [17] in which chemokine (i.e. CXCR1/2 agonist)-triggered opioid peptide release was less effectively blocked in rat compared to human neutrophils.

Opioid peptides can be readily detected in the inflamed synovial tissue of patients with arthritis [63] as well as in surgical wound [64]. Local opioid-mediated analgesia significantly reduces postoperative pain in humans since intraarticular naloxone administration enhances pain and consumption of pain medication, indicating a continuous release of opioid peptides [19],[65]. In the present study in rats, we delineated a molecular pathway of tonic opioid release from neutrophils in complete Freund's adjuvant-induced inflammation involving mycobacterially triggered FPR activation. Mycobacteria or bacterial products may trigger opioid peptide release in arthritic joints or at the site of surgery with accompanying infection. In addition, formyl peptides can also be released from mitochondria of eukaryotes [66]–[68]. Alternatively, other releasing agents such as chemokines (CXCR1/2 ligands) can trigger opioid peptide release from rat and human neutrophils [17],[18] and these are produced in complete Freund's adjuvant-induced inflammation [14] as well as in surgical wounds [69].

## Materials and Methods

### Antibodies and reagents

Rabbit anti-Met-enkephalin or anti-rat-β-endorphin Abs as well as purified Met-enkephalin and Boc-FLFLF were purchased from Bachem, Weil am Rhein, Germany. Naloxone, D-Phe-Cys-Tyr-D-Trp-Orn-Thr-Pen-Thr-NH_2_ (CTOP), naltrindole hydrochloride (NTI) and fMLP were obtained from Sigma-Aldrich Chemie, Deisenhofen, Germany, and desiccated *Mycobacterium butyricum* was from BD Bioscience, Heidelberg, Germany. Complete Freund's adjuvant, LY294002, wortmannin and 1,2-*bis*(*o*-aminophenoxy)ethane-N,N,N′,N′-tetraacetic acid acetoxymethyl ester (BAPTA/AM) were purchased from Calbiochem, San Diego, CA, USA. BAPTA/AM, LY294002, wortmannin, fMLP, and Boc-FLFLF were dissolved in dimethyl sulfoxide (maximal final concentration 1%). Anti-neutrophil serum was obtained from Accurate Chemical&Scientific Corporation, Westbury, NY, USA. Cyclosporine H was purchased from Eton Bioscience, San Diego, CA, USA. Anti-TLR-2-phycoerythrin (PE, clone TL2.1) and anti-TLR-4-PE (clone HTA125) as well as mouse IgG_2a_ were obtained from eBioscience, San Diego, CA, USA. Anti-TLR-2 (clone TL2.1) and anti-TLR-4 (clone HTA125) were from Alexis, Lörrach, Germany. fMLP-fluorescein isothiocyanate (FITC) was obtained from Invitrogen-Molecular Probes, Karlsruhe, Germany. CD45-CyC, RP-1-PE and ED1-PE were obtained by BD Biosciences, Heidelberg, Germany and Serotec, London, Great Britain, respectively

### Animals and complete Freund's adjuvant-induced inflammation

Male Wistar rats weighing 180–220 g were injected intraplantarly with 150 µl complete Freund's adjuvant in the right hind paw as described [70]. Experiments were conducted at 2–24 h after inoculation. All injections were performed under brief isoflurane anesthesia. Animal protocols were approved by the animal care committee of local authorities and were in accordance with the guidelines of the International Association for the Study of Pain [71].

### Measurement of hyperalgesia and analgesia

Mechanical nociceptive thresholds were assessed using the paw pressure algesiometer (modified Randall-Selitto test; Ugo Basile) as described before [14],[17]. The pressure required to elicit paw withdrawal using a blunt piston onto the dorsal surface of the hind paw, the paw pressure threshold, was determined. The treatments were randomized and the experimenter was blinded to the treatments. A decrease in the paw pressure threshold was interpreted as hyperalgesia (pain) whereas a rise in the paw pressure threshold was interpreted as analgesia (antinociception).

Thermal nociceptive thresholds were measured by the Hargreaves test [42]. The latency (time; s) required to elicit paw withdrawal was measured with an electronic timer (IITC Inc/Life Science) after application of radiant heat to the plantar surface of a hind paw from underneath the glass floor with a high-intensity light bulb. The stimulus intensity was adjusted to give 20 s paw withdrawal latency in noninflamed paws, and the cutoff was 25 s to avoid tissue damage. The average of two measurements taken with 20 s intervals was calculated. A decrease in paw withdrawal latency was interpreted as pain (hyperalgesia) whereas a rise in paw withdrawal latency was interpreted as analgesia (antinociception).

### Experimental protocols

fMLP-induced analgesia was evaluated in rats with complete Freund's adjuvant inflammation after intraplantar (i.pl.) injection of 0.1–3 ng fMLP dissolved in 100 µl of NaCl 0.9% or of solvent only. Paw pressure threshold or paw withdrawal latency were measured 5 min after fMLP injection. In some experiments the opioid receptor antagonist naloxone (0.56 ng i.pl.), CTOP (2 µg i.pl.), NTI (50 µg i.pl.), or an antibody against Met-enkephalin (1.25 µg i.pl.) were injected concomitantly. Optimal doses were determined in pilot experiments and in previous studies [17],[18]. To deplete rats of neutrophils, animals were injected with 80 µl anti-neutrophil serum intravenously 18 h before complete Freund's adjuvant injection as described previously [14],[17].

Modulation of baseline inflammatory thermal hyperalgesia was analyzed in rats with complete Freund's adjuvant (2–24 h) inflammation after i.pl. treatment with naloxone (0.56 ng), anti-Met-enkephalin (1.25 µg), anti-β-endorphin (2 µg), FPR antagonists (Boc-FLFLF: 0.3 and 3 µg or cyclosporine H: 0.9 and 9 µg) or after systemic neutrophil depletion.

### Monocyte isolation by MACS separation

To obtain human monocytes from healthy blood donors, red blood cells were lyzed using buffer EL (Qiagen, Hilden, Germany). The remaining white blood cells were incubated with anti-CD14-coupled magnetic beads (Miltenyi Biotec, Bergisch-Gladbach, Germany) in phosphate-buffered saline (PBS) containing 0.5% bovine serum albumin and 2 mM ethylene diamine tetraacetic acid. CD14^+^ monocytes were isolated using a LS column (Miltenyi Biotec). Purity was confirmed by staining with anti-CD14 FITC antibody (BD Biosciences) and FACS analysis (see below).

### Opioid peptide release

Human neutrophils from healthy blood donors were purified using dextran sedimentation, Ficoll separation and hypotonic lysis (all Amersham Biosciences). Rat peritoneal neutrophils were obtained 4 h after intraperitoneal injection of 1% oyster glycogen (Sigma-Aldrich Chemie) [17],[72].

For determination of opioid peptide release, 5×10^7^ neutrophils or 1×10^7^ CD14^+^ monocytes were stimulated with fMLP (1–1000 nM) or *Mycobacterium butyricum* (0.06–0.66 mg/ml) after preincubation with cytochalasin B (5 µg/ml) for 5 min in Hank's balanced salt solution containing the proteinase inhibitors bestatin (5 µg/ml), aprotinin (40 µg/ml) and thiorphan (100 µM, all Sigma-Aldrich Chemie) [17],[73],[74]. Doses of fMLP and *Mycobacterium butyricum* were based on pilot experiments and the literature [74],[75]. In some experiments, cells were concomitantly incubated with inhibitors as described in the results section. Doses were established in pilot experiments and according to the literature [17],[31],[35]. Control samples with the solvent dimethyl sulfoxide did not induce significant release. Doses for anti-TLR-2 (clone TL2.1) and anti-TLR-4 (clone HTA125) antibodies were chosen based on their blocking effects according to the literature [46], [49]–[52]. Supernatants were obtained after 7 min stimulation and stored at −20°C until further analysis by radioimmunoassay using commercially available kits for rat or human Met-enkephalin and β-endorphin (Bachem) [17],[76],[77].

### Cell culture and transfection

Construction of plasmids coding for the human fMLP receptor has been described elsewhere [78]. HEK293 cells were grown at 37°C and 5% CO_2_ in Dulbecco's modified Eagle's medium or minimal essential medium with Earle's salts, supplemented with 10% fetal calf serum, 2 mM glutamine, 100 µg/ml streptomycin, and 100 units/ml penicillin. HEK293 cells were transfected using Fugene 6 transfection reagent (Roche Applied Science, Mannheim, Germany) according to the manufacturer's recommendations. The amount of transfected human FPR plasmid cDNA was 250 ng per 35 mm dish and was kept constant by addition of empty expression vector (pcDNA3 up to 2 µg) where necessary.

### Ca^2+^ imaging

Fluorescence imaging was performed with a monochromator-equipped xenon lamp and a cooled CCD camera (TILL-Photonics) connected to an inverted epifluorescence microscope (Axiovert 100; Carl Zeiss). All imaging experiments were performed in a Hepes-buffered solution containing 128 mM NaCl, 6 mM KCl, 1 mM MgCl_2_, 1 mM CaCl_2_, 5.5 mM glucose, 10 mM Hepes (pH 7.4), and 0.2% (wt/vol) bovine serum albumin. For determination of [Ca^2+^]*_i_*, neutrophils or FPR transfected HEK293 cells were placed on dishes coated with poly-L-lysine and then loaded with 1 or 2 µM Fura 2/AM (Molecular Probes-Invitrogen) for 30 min at 37°C as previously described [79]. After basal recordings, cells were stimulated by subsequent addition of 1 µM fMLP or dimethyl sulfoxide extracted *Mycobacterium butyricum*. Fura-2 loaded cells were alternately excited at 340 and 380 nm, and fluorescence was detected through a 505 nm filter. Calibration of [Ca^2+^]*_i_* was performed as described [17],[79].

### Flow cytometry

TLRs were labeled in human neutrophils after preincubation with 10% mouse serum for 10 min using PE conjugated anti-human-TLR-2, anti-human-TLR-4 or isotype control antibodies according to manufacturer's instructions. The FPR was stained using FITC-conjugated fMLP (1 µM). In selected experiments samples were pretreated for 10 min with different concentrations of Boc-FLFLF before addition of FITC-fMLP [13],[14],[17],[76].

FPR staining in subcutaneous paw tissue was performed as described before [17]. Neutrophils were identified by CD45^+^ and RP1^+^ staining while macrophages were CD45^+^ ED1^+^.

### Immunofluorescence

Immunofluorescence staining was performed using human neutrophils (5 min preincubation with cytochalasin B, then addition of 0.66 mg/ml *Mycobacterium butyricum* for 15 min) [41] as well as neutrophils preincubated with Boc-FLFLF. After centrifugation for 10 min at 300 g, cell pellets of neutrophils were reconstituted in 5 ml PBS, and 50,000 neutrophils in suspension were then centrifuged by a Shandon Cytospin 3 (Thermo Shandon, Pittsburgh, PA) at 20 g for 3 min on glass slides. Neutrophils were fixed for 30 min and confocal analysis was carried out as previously described [80]. Briefly, neutrophil cytospins were incubated with rabbit polyclonal antibodies against Met-enkephalin (1∶1000, Peninsula Laboratories, Belmont, CA, USA) and subsequently with a Texas red-conjugated goat anti-rabbit antibody. Thereafter, cytospins were washed with PBS and mounted in vectashield. Images were acquired on a Zeiss LSM510META confocal laser scanning system (Zeiss AIM; Jena) using a 63×/1.4 Plan-Apochromat or 40×/1.3Plan-Neofluar oil immersion objective in a series of optical sections of about 1 µm thickness. Each experiment was repeated three times. To demonstrate specificity of staining, the following controls were included as mentioned in detail elsewhere [14],[81]: (1) preabsorption of diluted antibody against Met-enkephalin with purified Met-enkephalin (Peninsula laboratories-Bachem) and (2) omission of either the primary or the secondary antibodies.

### Statistical analysis

Data are presented as raw values (mean±SEM). Normally distributed data were analyzed by student's t-test or Mann-Whitney test. Not normally distributed data were analyzed by Wilcoxon Signed Rank Test. Multiple comparisons were analyzed by one-way ANOVA or by one-way ANOVA on ranks in case of not normally distributed data. If necessary repeated measures (RM) one way ANOVA was used. Posthoc comparisons were performed by Student-Newman-Keuls', Dunnett's or Duncan's method, respectively. Differences were considered significant if p<0.05. Dose dependency was evaluated by linear regression analysis. Sigma Stat was used to analyze the data.

## Supporting Information

Figure S1 β-Endorphin release from neutrophils is triggered by mycobacteria and FPR agonists but not by toll like receptor-2 or toll like receptor-4 agonists. [A–C] Rat and human neutrophils as well as CD14^+^ human monocytes were incubated with heat-inactivated *Mycobacterium butyricum* (*Myco. but.*), and β-endorphin (END) release was quantified by radioimmunoassay (n = 5–12 * p<0.05, one way RM ANOVA, Student-Newman-Keuls Method). [D–F] Human (n = 8–14) and [G–I] rat neutrophils (n = 5–10) were stimulated with the TLR-2 agonist peptidoglycan, the TLR-4 agonist lipopolysaccharide or the FPR agonist fMLP, and Met β-endorphin (END) release was measured in the supernatant (* p<0.05; one way RM ANOVA, Student-Newman-Keuls Method). Data are presented as means+/−SEM.
(0.26 MB TIF)
