# Supplementary figures and images for: Mycobacteria Attenuate Nociceptive Responses by Formyl Peptide Receptor Triggered Opioid Peptide Release from Neutrophils
Source: PLoS Pathog. 2009 Apr 3;5(4):e1000362. doi: 10.1371/journal.ppat.1000362 (PMC2657213; doi:10.1371/journal.ppat.1000362)

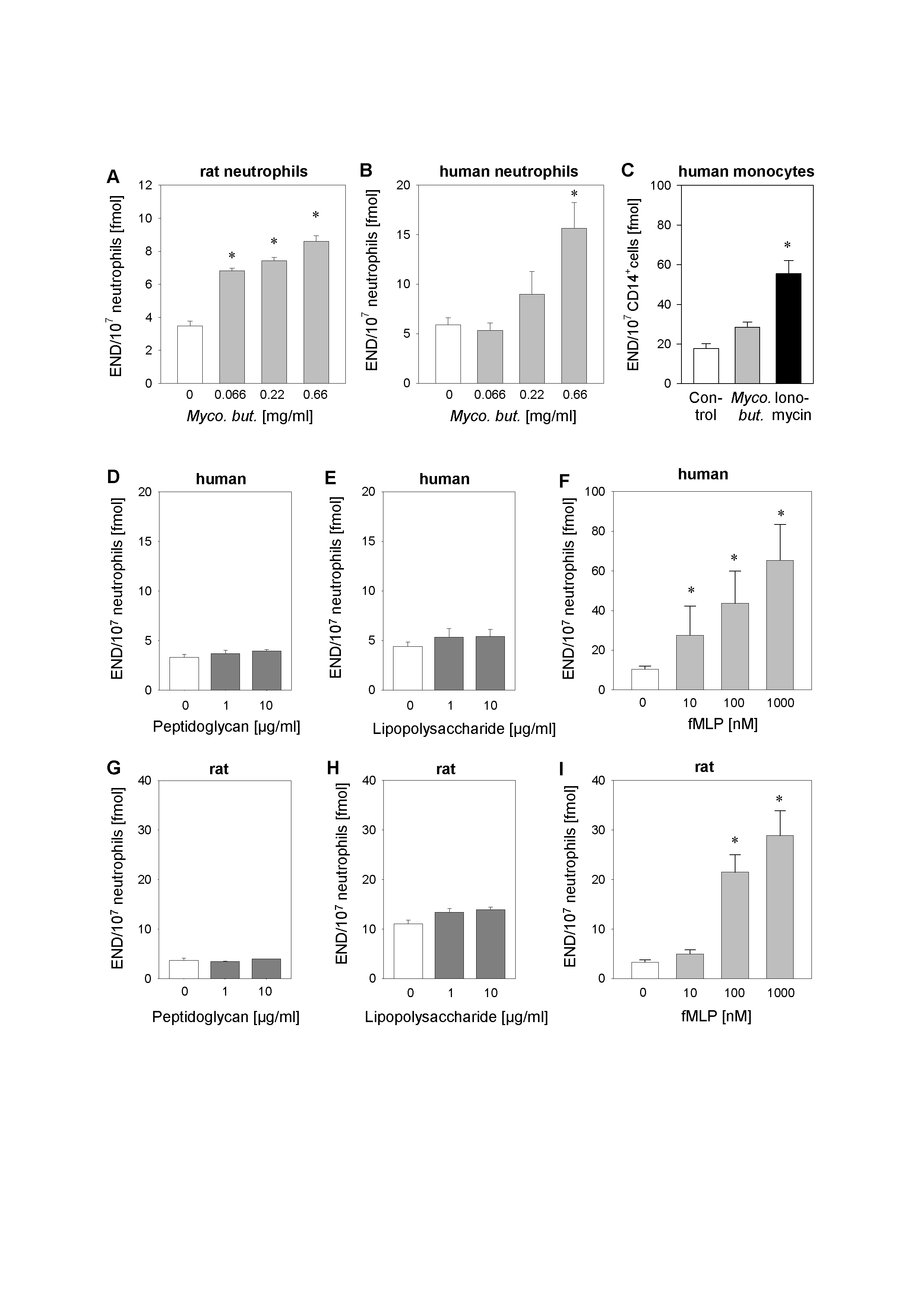

Supplement: Figure S1 — β-Endorphin release from neutrophils is triggered by mycobacteria and FPR agonists but not by toll like receptor-2 or toll like receptor-4 agonists. [A–C] Rat and human neutrophils as well as CD14+ human monocytes were incubated with heat-inactivated Mycobacterium butyricum (Myco. but.), and β-endorphin (END) release was quantified by radioimmunoassay (n = 5–12 * p<0.05, one way RM ANOVA, Student-Newman-Keuls Method). [D–F] Human (n = 8–14) and [G–I] rat neutrophils (n = 5–10) were stimulated with the TLR-2 agonist peptidoglycan, the TLR-4 agonist lipopolysaccharide or the FPR agonist fMLP, and Met β-endorphin (END) release was measured in the supernatant (* p<0.05; one way RM ANOVA, Student-Newman-Keuls Method). Data are presented as means+/−SEM. (0.26 MB TIF) [file ppat.1000362.s001.tif]
